# ENJOYMENT OF DAILY ACTIVITIES IN OLDER CAREGIVERS: AN ECOLOGICAL MOMENTARY ASSESSMENT

**DOI:** 10.1093/geroni/igae098.0325

**Published:** 2024-12-31

**Authors:** Abel Pichardo, Brent Mausbach

**Affiliations:** San Diego State University, San Diego, California, United States; University of California San Diego, La Jolla, California, United States

## Abstract

Behavioral Activation (BA) theory posits that depression stems from poor exposure to positive environmental rewards, with increased positive activities linked to mood improvement. BA theory may explain the high prevalence of depressive symptoms observed in informal caregivers. The current study used an Ecological Momentary Assessment approach to investigate the association between daily variability in activities with variability in daily mood. Participants were 64 informal caregivers of Alzheimer’s Disease or related disorders. Using a digital diary, participants recorded daily their mood, number of activities, and enjoyment ranging from 2 to 335 days. Enjoyment of activities and daily mood were rated on scales ranging from 1 to 5 (1 being least pleasant). A linear mixed-model analysis examined the associations between variability in mood with variability in both frequency and enjoyment of daily activities. Results indicated that a greater number of activities was associated with more positive mood for the day (B=0.045, t=13.58, p<.001). Greater average enjoyment of activities was also associated with more positive mood for the day (B=0.5, t=33.40, p<.001). There was a significant interaction of total activities by average enjoyment (B=-0.02, t=-8.49, p<.001). Post-hoc analyses indicated that increasing activation by 8 daily activities indicated an improvement in mood of approximately 0.3 points. However, replacing two moderately enjoyable activities with highly enjoyed activities indicated an improvement in mood of nearly 0.7 points. These findings suggest that modifying types of activities to focus on enjoyment rather than total activity may provide greater impact to overall mood.

